# Development and evaluation of *Fusarium* wilt‐resistant and high‐yielding chickpea advanced breeding line, KCD 11

**DOI:** 10.1002/tpg2.20460

**Published:** 2024-05-21

**Authors:** C. Laxuman, Yogesh Dashrath Naik, B. K. Desai, Mallikarjun Kenganal, Bharat Patil, B. S. Reddy, D. H. Patil, Sidramappa Chakurte, P. H. Kuchanur, Shiva Kumar K, Ashok Kumar Gaddi, L. N. Yogesh, Jayaprakash Nidagundi, B. M. Dodamani, Gururaj Sunkad, Mahendar Thudi, Rajeev K. Varshney

**Affiliations:** ^1^ Zonal Agricultural Research Station (ZARS), Kalaburagi University of Agricultural Sciences‐Raichur (UAS‐R) Raichur Karnataka India; ^2^ Department of Agricultural Biotechnology and Molecular Biology Dr. Rajendra Prasad Central Agricultural University (RPCAU) Samastipur Bihar India; ^3^ Department of Agronomy University of Agricultural Sciences Raichur Karnataka India; ^4^ Department of Genetics and Plant Breeding Agricultural Research Station, UAS‐R Bidar Karnataka India; ^5^ Department of Genetics and Plant Breeding Collage of Agriculture, UAS‐R Bheemarayanagudi Karnataka India; ^6^ Department of Genetics and Plant Breeding Main Agricultural Research Station (MARS), UAS‐R Raichur Karnataka India; ^7^ Department of Soil Science Agricultural Research Station, Sirguppa, UAS‐R Sirguppa Karnataka India; ^8^ Department of Genetics and Plant Breeding Agricultural Research Station, UAS‐R Hagari Karnataka India; ^9^ Department of Plant Pathology University of Agricultural Sciences Raichur Karnataka India; ^10^ Centre for Crop Health and School of Agriculture and Environmental Science University of Southern Queensland (UniSQ) Toowoomba Queensland Australia; ^11^ College of Agriculture, Family Sciences and Technology Fort Valley State University Fort Valley Georgia USA; ^12^ Center of Excellence in Genomics & Systems Biology International Crops Research Institute for the Semi‐Arid Tropics (ICRISAT) Patancheru Telangana India; ^13^ WA State Agricultural Biotechnology Centre, Centre for Crop and Food Innovation Murdoch University Murdoch Western Australia Australia

## Abstract

*Fusarium* wilt (FW) is the most severe soil‐borne disease of chickpea that causes yield losses up to 100%. To improve FW resistance in JG 11, a high‐yielding variety that became susceptible to FW, we used WR 315 as the donor parent and followed the pedigree breeding method. Based on disease resistance and yield performance, four lines were evaluated in station trials during 2017–2018 and 2018–2019 at Kalaburagi, India. Further, two lines, namely, Kalaburagi chickpea desi 5 (KCD 5) and KCD 11, which possesses the resistance allele for a specific single‐nucleotide polymorphism marker linked with FW resistance, were evaluated across six different locations (Bidar, Kalaburagi, Raichur, Siruguppa, Bhimarayanagudi and Hagari) over a span of 3 years (2020–2021, 2021–2022 and 2022–2023). KCD 11 exhibited notable performance, showcasing yield advantages of 8.67%, 11.26% and 23.88% over JG 11, and the regional checks Super Annigeri 1 (SA 1) and Annigeri 1, respectively, with enhanced FW resistance in wilt sick plot. Further, KCD 11 outperformed JG 11, SA 1 and Annigeri 1 in multi‐location trials conducted across three seasons in the North Eastern Transition Zone, North Eastern Dry Zone, and North Dry Zones of Karnataka. KCD 11 was also tested in trials conducted by All India Coordinated Research Project on chickpea and was also nominated for state varietal trials for its release as a FW‐resistant and high‐yielding variety. The selected line is anticipated to cater the needs of chickpea growers with the dual advantage of yield increment and disease resistance.

## Core Ideas

- Pedigree breeding was used to develop Kalaburagi chickpea desi (KCD) 11, a *Fusarium* wilt (FW) resistant and high‐yielding advanced breeding line.
- KCD 11 recorded 8.67%, 11.26%, and 23.88% yield advantages over JG 11, Super Annigeri 1 (SA 1), and Annigeri 1, respectively.
- On an average KCD 11 has 14.20% and 13.90% higher yield over JG 11 and SA 1, respectively.
- The presence of resistant alleles in KCD 11 for single‐nucleotide polymorphism (SNP) marker (FW2_30366110) linked to FW further confirms its resistance.

**Abbreviations**
AICRP: All India Coordinated Research Project
AVT: advanced varietal trial
FW: *Fusarium* wilt
IVT: initial varietal trial
KCD: Kalaburagi chickpea desi
MLT: multi‐location trial
NEPZ: north eastern plain zone
SNP: single‐nucleotide polymorphism
ST: station trial
SZ: south zone

## 1 INTRODUCTION

Chickpea (*Cicer arietinum* L.) is one of the most important legume crop in the world. It is a self‐pollinated crop with a genome size of 738 Mb (Varshney et al., 2013) and is mostly cultivated on residual soil moisture during the winter season. It is cultivated in approximately 50 nations across the globe on diverse soil types and agro‐climatic conditions. Over the last several years, India has been the top producer of chickpea, with a global annual production of ∼11.91 million tonnes from an area of ∼10.94 million ha, with an average yield of 1.09 tonnes/ha (FAOSTAT, 2021). Chickpea consumption is popular in many regions across the globe, mainly due to its high nutritional quality, which provides the major source of dietary protein and is also being used for its nutraceutical and prebiotic properties (Mathew et al., 2022). Additionally, it plays an important role in crop rotation, mixed cropping and intercropping to balance soil fertility through nitrogen fixation and the release of soil‐bound phosphorus (Khan et al., 2020).

Unpredictable variations in the duration and severity of some extreme weather conditions and climate change are the major issues that have negative impact on chickpea production. These abiotic stresses can alter plant pathogen interactions by making the host plant more vulnerable to pathogen infection and insect attack (Pandey et al., 2017). *Fusarium* wilt (FW), caused by the pathogen *Fusarium oxysporum* f.sp*. ciceris*, is a critical soil‐borne disease and a primary factor leading to the underperformance of high‐yielding chickpea cultivars. Previous studies have reported that FW can destroy chickpea, causing 60%–100% yield losses (Halila & Strange, 1997; Nathawat et al., 2017). These biotic and abiotic stresses are predicted to create more hurdles in the coming days. To maintain food security and safety in the upcoming years, it is necessary to address these emerging challenges and work toward the development of crop varieties that possess both biotic and abiotic stress tolerance.

Till date, more than 350 improved chickpea cultivars have been developed through conventional breeding methods with improved yield, productivity, and adaptibility to new niches (Gaur et al., 2012). The high‐yielding cultivars such as Kranti (ICCC 37), Bharati (ICCCV 10), JG 11, Phule G 95311, and Super Annigeri 1 (SA 1) are recommended for cultivation in south India. Additionally, they have been utilized in several breeding initiatives to create breeding stock or high‐yielding stress tolerant chickpea cultivers. JG 11 is an elite, high‐yielding desi chickpea variety, which is extensively cultivated across India. It also has salinity‐tolerant properties and has been used to develop a mapping population for identifying genomic regions associated with salinity tolerance (Pushpavalli et al., 2015). Deployment of host resistance is the most preferred strategy for managing FW in chickpea, considering its long‐term benefits, minimal environmental impact, and cost‐effectiveness. Cultivars like Vijay and WR 315 have been used as donor parents for FW resistance against race 2 (*Foc 2*) and 4 (*Foc 4*) pathotypes, respectively (Mannur et al., 2019; Pratap et al., 2017). Marker‐assisted breeding has been widely used in breeding programs aimed toward improving disease resistance as well as drought tolerance in chickpea (Bharadwaj et al., 2022; Mannur et al., 2019; Varshney et al., 2014). All these studies suggested the importance of the high‐yielding and stress‐tolerant varieties as a source of breeding material for further chickpea improvement. Advancements in genomics have accelerated plant breeding programs aimed at developing crops that can withstand climatic challenges. However, traditional breeding methods still hold potential, and numerous initiatives pursuing this approach are underway worldwide.

In the past, we employed various chickpea genotypes and breeding lines to assess their yield response across diverse environments. From this, we selected chickpea lines that exhibited favorable performance concerning both overall yield and their ability to thrive in diverse environments (Laxuman et al., 2022). In the present study, we report the use of a pedigree method, which enabled the development of a FW resistant and high‐yielding line Kalaburagi chickpea desi 11 (KCD 11).

## 2 MATERIALS AND METHODS

### 2.1 Plant material

In order to develop high‐yielding FW resistant chickpea lines, we chose WR 315 as the donor and JG 11 as the recipient parental lines and deployed a pedigree‐based breeding method. WR 315 is a landrace that is known for its FW resistance allele race 4 (*Foc 4*), while JG 11 (ICCV 93954) is a desi chickpea variety with early maturity (95–100 days) and high‐yielding properties. Also, JG 11 is a FW resistant chickpea variety that was developed and released in 1999 for cultivation in the southern zone (Orrisa, Karnataka, Andhra Pradesh and Tamil Nadu).

**Core Ideas**

- Pedigree breeding was used to develop Kalaburagi chickpea desi (KCD) 11, a *Fusarium* wilt (FW) resistant and high‐yielding advanced breeding line.
- KCD 11 recorded 8.67%, 11.26%, and 23.88% yield advantages over JG 11, Super Annigeri 1 (SA 1), and Annigeri 1, respectively.
- On an average KCD 11 has 14.20% and 13.90% higher yield over JG 11 and SA 1, respectively.
- The presence of resistant alleles in KCD 11 for single‐nucleotide polymorphism (SNP) marker (FW2_30366110) linked to FW further confirms its resistance.

### 2.2 Development and evaluation of FW resistant lines in sick plot

**FIGURE 1:**
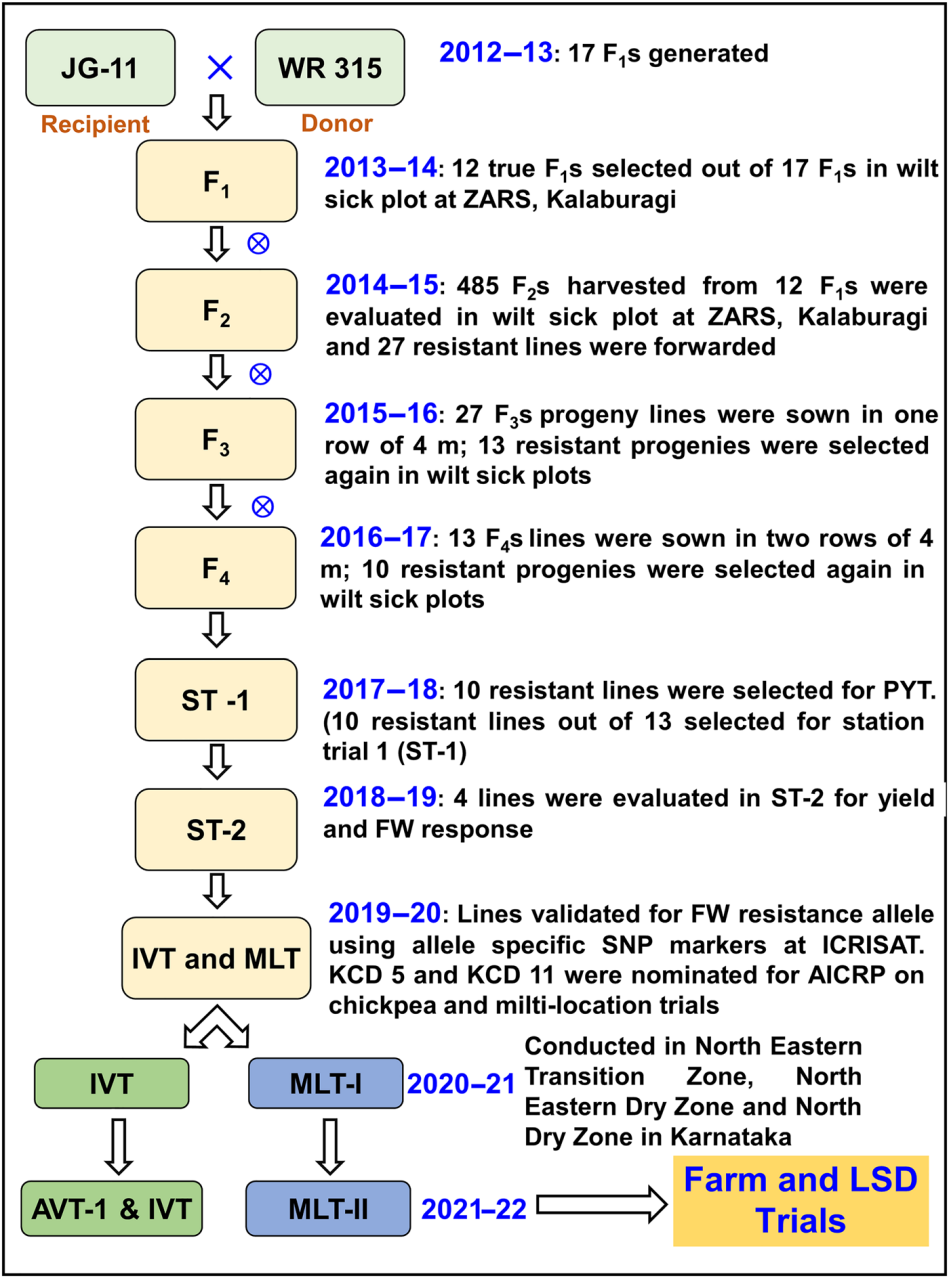
A schematic representation of the development of *Fusarium* wilt (FW) resistant and high‐yielding chickpea line, Kalaburagi chickpea desi 11 (KCD 11). The breeding process involved the utilization of JG 11 (recipient) and WR 315 (donor) as parent lines, and the approach employed was the pedigree method. In each generation, FW resistant and high‐yielding lines were selected by screening the lines in wilt sick plot. AVT, advance varietal trial; IVT, initial varietal trial; MLT, multiple‐location trial; **⊗**, self‐pollination/selfing.

Development of a FW resistant and high‐yielding line, KCD 11, using pedigree selection is schematically represented in Figure 1. During the cropping season of 2012–2013, crosses were made at the Agricultural Research Station (ARS) Kalaburagi, Karnataka, India (17.362252° N, 76.816358° E) between JG 11 and WR 315, resulting in the acquisition of 17 F_1_ seeds. In brief, the resistant lines obtained from each generation were advanced to the next generation in a wilt sick plot at Zonal Agricultural Research Station (ZARS), Kalaburagi, Karnataka (Figure 1). We screened the newly developed chickpea lines along with local promising chickpea varieties (Annigeri 1, SA 1, JG 11, JG 62 and WR 315) in FW sick plot at ZARS, Kalaburagi, Karnataka. A wilt‐susceptible check (JG‐62) was randomly sown in plots to monitor disease pressure in chickpea. The pathogen load in the sick plot was enriched over successive crop seasons by adding more pathogen culture in plot. The percentage of wilt incidence was calculated based on the initial plant count and the total number of wilted plants (Irulappan et al., 2021).

### 2.3 Station and multi‐location field trials

A total of four resistant lines along with nine advanced breeding lines were evaluated at the ZARS, Kalaburagi Research fields during 2017–2018 and 2018–2019. These evaluations were called as station trial‐I (ST‐I) and ST‐II, respectively. The lines were evaluated in wilt sick plots along with JG 11 (national check) and SA 1 (zonal check), following complete randomized block design (RCBD) in four rows of 4 m long beds. Further, two lines, namely, KCD 11 and KCD 5, selected based on their disease reaction and yield performance in ST‐I and ST‐II, were evaluated in six locations (Bidar, Raichur, Siruguppa, Hagari, Bheemarayana Gudi and Kalaburagi), along with National check (JG 11) and zonal check (SA 1) during the cropping seasons of 2020–2021, 2021–2022 and 2022–2023. Multi‐location trials (MLTs) I and II were conducted using complete RCBD, and the seeds of these lines were sown in four rows of 4 m long beds.

### 2.4 Validation for the presence of FW resistance alleles

We validated the selected lines for the presence of FW resistance alleles using single‐nucleotide polymorphism (SNP) markers reported earlier (Palakurthi et al., 2021). In brief, genomic DNA from the four chosen lines was isolated from healthy young leaves using mini‐DNA extraction protocol (Manchikatla et al., 2021). The quality of the extracted genomic DNA was evaluated via gel electrophoresis on a 1% agarose gel, and its concentration was measured using the NanoDrop 2000 spectrophotometer. Subsequently, the DNA was diluted with double‐distilled water to a concentration of 20 ng/mL and employed as the template for PCR amplification.

### 2.5 All India Coordinated Research Projects (AICRP) on chickpea trials

After confirming the presence of FW resistance alleles through marker validation, the lines KCD 11 and KCD 5 were nominated for AICRP on chickpea for evaluating their performance in initial varietal trials (IVT) during 2020–2021. Further, these lines were also evaluated under IVT‐irrigated timely sown and IVT‐rainfed timely sown conditions in five locations, along with checks during 2021–2022 crop season in the South Zone. In addition, these lines were promoted to advanced varietal trials (AVT) conducted in north eastern plain zone (NEPZ) during crop season 2021–2022.

## 3 RESULTS AND DISCUSSION

In general, creation of genetic variability, selection of desired phenotypes, and subsequent evaluation of selected lines represent three basic steps in most of the breeding programs. In the case of chickpea, methods such as earlier pedigree, bulk, modified bulk‐pedigree, back‐cross, single seed descent (SSD), selective random mating, and mutation breeding have been used for crop improvement. Based on earlier reports from ICRISAT and the breeding programs at ZARS Kalaburagi, we found pedigree, modified bulk, and SSD methods to be comparatively more useful for developing advanced breeding lines. *Fusarium oxysporum* f.sp. *ciceris* causes FW by clogging the xylem vessels within the host plant (Priyadarshini et al., 2023). This obstruction disrupts the normal water transport inside the plant, ultimately leading to wilting and the complete collapse of the plant onto the ground. The damage due to FW, particularly from race 1 is prevalent in southern states of India where warmer climates prevail. In order to develop FW resistant and high‐yielding chickpea advanced breeding line, we adopted pedigree breeding followed by confirmation of resistance alleles in the developed lines using SNP markers.

### 3.1 Development of FW resistant lines

Development of a FW resistant and high‐yielding line, KCD 11, using pedigree selection is schematically represented in Figure 1. Earlier, four kabuli chickpea genotypes resistant to FW, including ICCV 2, ICCV 3, ICCV 4 and ICCV 5, were developed using the pedigree method (see Yadav et al., 2023). Globally, several sources were identified and used as donors for enhancing FW resistance in chickpea (see Yadav et al., 2023). For instance, WR 315 (Mannur et al.,^2019^), JG 11, and Pusa 372 (Jorben et al., 2023) represent some notable sources of FW resistance. During recent years, WR 315 was extensively used as the donor for enhancing FW using marker‐assisted backcross breeding programs (Bharadwaj et al., 2022; Mannur et al., 2019; Pratap et al., 2017). In the present study, we used WR 315 as the donor parent. A total of 17 F_1_s were harvested from the cross between JG 11 and WR 315. During the 2013–2014 crop season, 12 true F_1_s were selected out of 17 based on their performance in the wilt sick plot. Subsequently, 485 F_2_ seeds were sown, which were harvested from the 12 selected F_1_s. Among these, 27 plants were further chosen based on their performance in wilt sick plot at Kalaburagi during crop season 2014–2015. During 2015–2016, 13 resistant F_3_s were selected based on their resistance to FW in wilt sick plot. Further, during 2016–2017, 13 F_4_ lines were sown in wilt sick plot in two rows of 4 m, and 10 resistant lines (KCD 5, KCD 11, KCD 55, KCD 20, KCD 24, KCD 25, KCD 26, KCD 56, KCD 19 and KCD 54) were subsequently selected.

### 3.2 Performance of developed lines in STs

**FIGURE 2:**
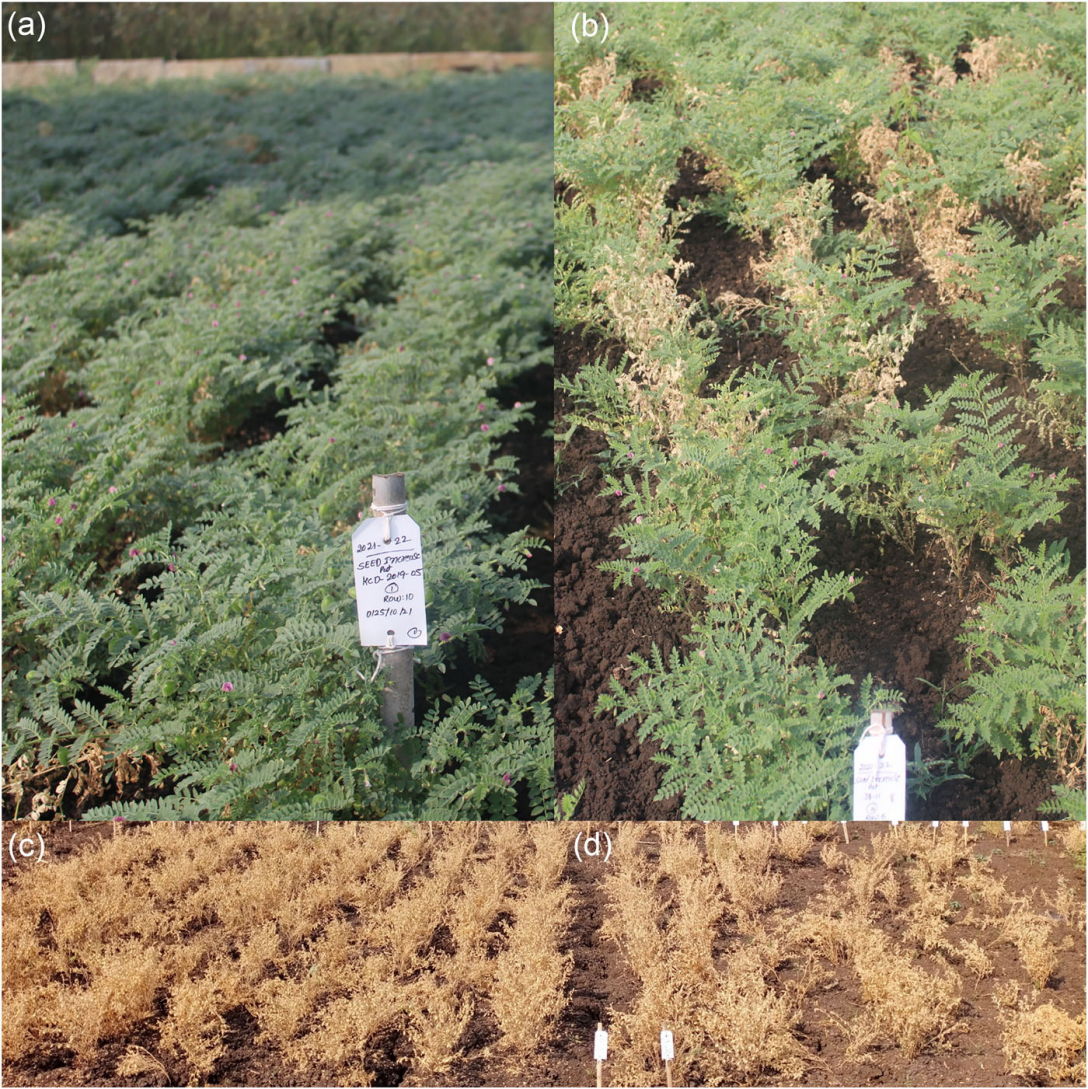
Evaluation of the performance of KCD 11 in wilt sick plots at Zonal Agricultural Research Station, Kalaburagi, during 2021–2022 season. At both the flowering stage and maturity, as depicted in panels (a) and (c) for KCD 11, and panels (b) and (d) for JG 11, the line KCD 11 exhibits improved resistance to *Fusarium* wilt compared to JG 11.

**FIGURE 3:**
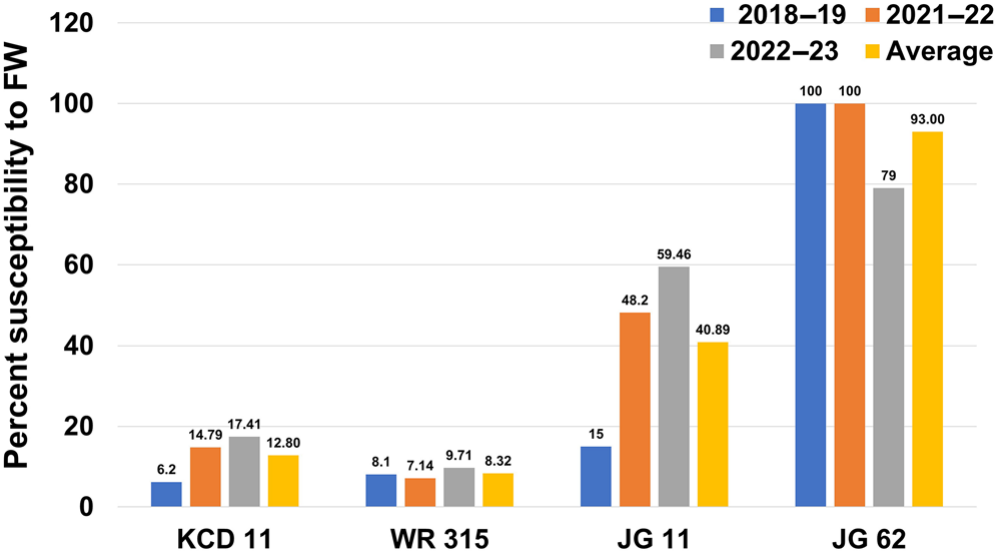
Disease reaction of chickpea advanced lines against *Fusarium* wilt. The bar plot represents the comparison between KCD 11and other local check varieties (WR 315, JG 11 and JG 62) evaluated for three seasons at Kalaburagi, Karnataka.

**FIGURE 4:**
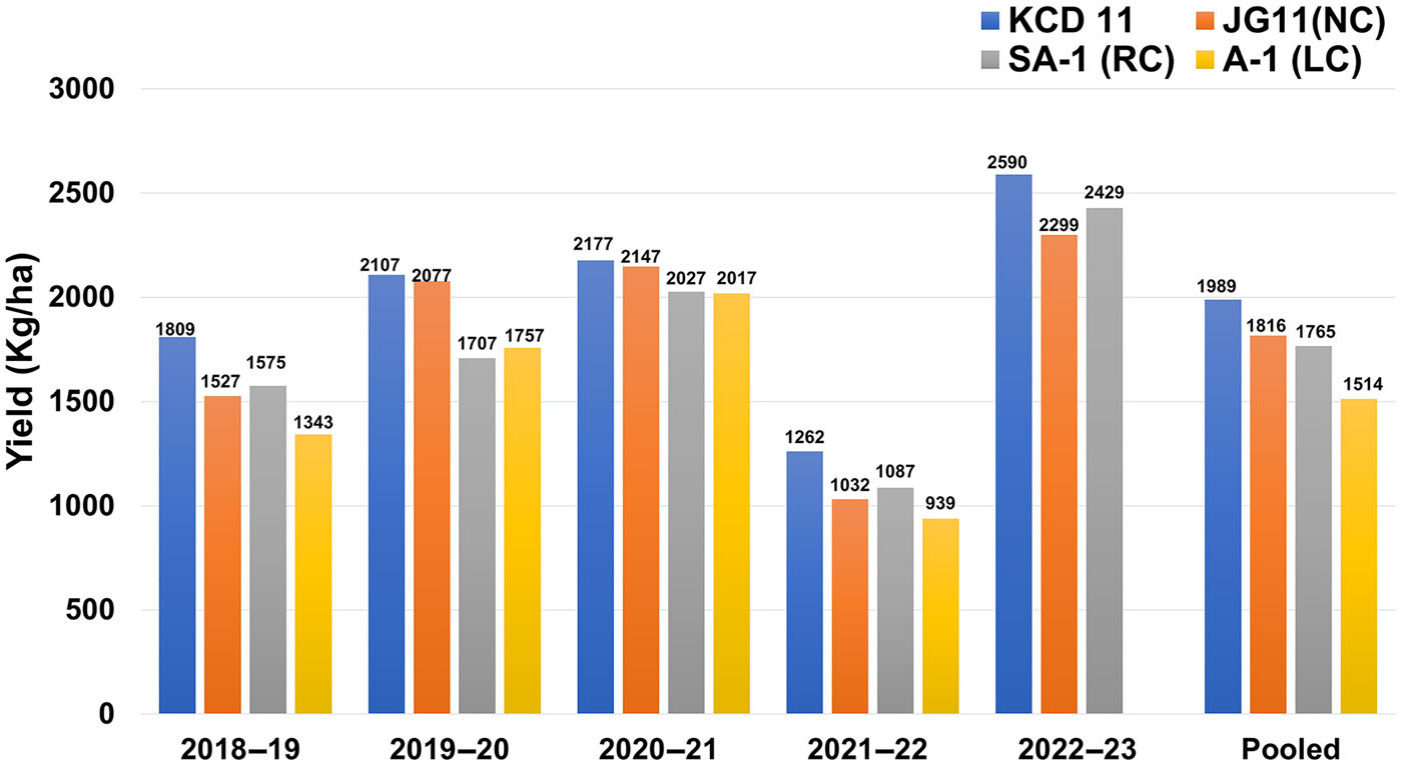
Yield performance of KCD 11 compared to national check (JG 11), regional check (SA‐1) and local check (Annigeri 1) varieties. Higher yield performance of KCD 11 was observed during all 5 years of evaluation.

During 2017–2018 crop season, ST‐I was conducted at Kalaburagi, specifically focusing on four resistant lines. Based on their resistance to FW and yield performance, four lines (KCD 5, KCD 11, KCD 55 and KCD 20) were selected for further assessment in ST‐II conducted during the 2018–2019 crop season (Table 1). Among them, two best performing lines, namely, KCD 5 and KCD 11, were further selected and evaluated in wilt sick plots at Kalaburagi for 3 years (2020–2021, 2021–2022 and 2022–2023), along with JG 11 and SA 1 (Table 2). A clear demarcation can be visualized from STs, revealing that KCD 11 possessed very low incidence of FW in the wilt sick plot both during early and late stages of the crop (Figure 2). During these evaluations, KCD 11 exhibited a moderate resistance reaction to FW, with susceptibility of 6.2%, 14.79% and 17.41% during crop seasons 2020–2021, 2021–2022 and 2022–2023, respectively (Figure 3). In contrast, JG 11 recorded 15%, 48.2% and 59.46% susceptibility to FW during crop seasons 2020–2021, 2021–2022 and 2022–2023, respectively (Figure 3). This rise in susceptibility occurred because the initial screening was done in a plot with a sickness level of 15%–18%. Later screenings were conducted in plots deliberately made more sick (69%–73%) by adding extra pathogen culture. So, initially, the reaction was resistant, but in enriched sick plot conditions, it became moderately resistant. Furthermore, WR 315 remained resistant to FW throughout the evaluations. These findings indicate that KCD 11 demonstrates a moderate resistance to FW, offering a valuable attribute in its disease resistance profile. In summary, the advanced breeding line, KCD 11, was evaluated for five seasons (2018–2019 to 2022–2023) at ZARS, Kalaburagi. The average yield across these seasons was determined to be 1989 kg/ha. Notably, KCD 11 displayed significant advantages in terms of yield compared to the checks SA 1 (11.26% advantage), Annigeri 1 (23.88% advantage), and JG 11 (8.67% advantage), as evidenced by their respective yield of 1765 kg/ha, 1514 kg/ha, and 1816.4 kg/ha, respectively (Figure 4).

**TABLE 1:** Disease reaction and yield performance of four lines in station trials conducted during 2017–2018 and 2018–2019.

Entry	2017–2018		2018–2019	
	FW occurrence (%)	Yield (kg/ha)	FW occurrence (%)	Yield (kg/ha)
KCD 20	4.3	2049	2.1	1143
KCD 55	5.8	2193	6.5	860
KCD 11	4.2	2468	6.2	1809
KCD 5	6.8	2384	4.7	1426
JG 11	17.0	2257	15.0	1493
WR 315	2.4	2036	3.3	1542
SA 1	7.1	2404	5.5	1638

Abbreviation: KCD, Kalaburagi chickpea desi.

**TABLE 2:** Yield (kg/ha) performance of KCD 11 at Zonal Agricultural Research Station (ZARS), Kalaburagi, across three seasons (2020–2021 to 2022–2023) and across five different sowing dates during 2022–2023.

Entry	Crop seasons			Sowing dates during 2022–2023				
	2020–2021	2021–2022	2022–2023	Oct. 9	Oct. 21	Nov. 5	Nov. 15	Dec. 5
KCD 11	2177	1262^a^	2590^a^	2779	1969	2015	1151^a^	839
JG 11	2147	1032	2299	2422	1750	1911	800	766
SA‐1	2027	1087	2429	2442	2306	1921	1231	555
CD 5%	406.57	215	215	505.54	497.26	379.96	229.29	185.55
CV %	11.66	13	5	13.15	17.22	14.25	13.74	16.72

^a^ Significant performance.

Besides timely sowings, farmers also cultivate chickpea by sowing late depending on the moisture availability. In order to assess suitability and yield performance of KCD 11 during crop season 2022–2023, we evaluated the advanced breeding line by sowing at five different time periods. Particularly, the crop was sown on October 9, October 21, November 5, November 15, and December 5 (Table 2). We performed these experiments with the assumption that the line KCD 11 developed in the genetic background of JG 11, the drought‐tolerant variety cultivated extensively in Karnataka, should perform better under late sown conditions that are prone to terminal drought and reproductive heat stress. Our experiments indicated that KCD 11 sown early (i.e., in the first week of October) recorded the highest yields. Nevertheless, the late sown (November 15) KCD 11 recorded significantly higher yield compared to JG 11 and SA 1. However, the crop sown in December did not show significant yield enhancement (Table 2). This indicates that KCD 11 can be recommended for late sowing under rainfed environments, until the mid of November.

### 3.3 Validation of FW associated allele‐specific primer

To identify resistant alleles in the developed advanced breeding line, a Polymerase chain reaction (PCR)‐based allele‐specific marker, FW2_30366110, developed at ICRISAT was used. PCR was performed using the DNA isolated from the 15 days old seedlings of KCD 11, JG 11 and WR 315 (Figure S1). Allele‐specific amplification was detected in the resistant parental line (WR 315) and KCD 11, while no allele‐specific amplification was observed in the case of the susceptible check. These genotyping results indicate the presence of the desired resistant alleles in KCD 11 and the selected advanced breeding lines, confirming the results obtained in the sick plot.

### 3.4 Performance of KCD 11 in multi‐location field trials

We conducted MLTs for three consecutive cropping seasons (2020–2021, 2021–2022 and 2022–2023) in three agroclimatic zones of Karnataka (India), namely, North Eastern Transition Zone (Zone I), North Eastern Dry Zone (Zone II) and North Dry Zone (Zone III). In total, we evaluated these lines in six locations, including one in Zone I (Bidar), three in Zone II (Kalaburagi, Bheemarayana Gudi and Raichur), and two in Zone III (Siruguppa and Hagari) (Table 3). Specifically, the overall yield performance of KCD 11 during 2020–2021, 2021–2022 and 2022–2023 was 1753.25, 1586.6, and 1950.40 kg/ha, respectively. Importantly, KCD 11 performed better than JG 11 in terms of yield in all three types of trials. On an average, we observed that KCD 11 recorded a yield advantage of 13.39% and 9.06% compared to JG 11 and SA 1, respectively. Similar trends were observed in Zone II and Zone III (Table 3). In the MLT, an average yield of 1803 kg/ha was recorded, which was 14.22% higher than JG 11. In field trials, KCD 11 produced an average yield of 1304 kg/ha, which was 20.74% higher than JG 11. In large‐scale trials, KCD 11 exhibited an average yield of 1633 kg/ha, marking a 22% increase compared to JG 11. These results indicate that KCD 11 stands out as a high‐yielding chickpea variety, surpassing JG 11 across different agro‐climatic conditions. The outstanding performance displayed by KCD 11 throughout all the trials positions it as a favorable option for farmers who are seeking alternatives to JG 11 and other cultivars grown in the area.

**TABLE 3:** Summary of yield (kg/ha) performance of KCD 5 and KCD 11 in the North Eastern Transition Zone, North Eastern Dry Zone, and North Dry Zone in Karnataka.

Entry	2020–2021				2021–2022					2022–2023				
	E1	E2	E3	E4	E1	E2	E3	E5	E6	E1	E2	E3	E5	E6
KCD 5	1486	2078	1062	2343	1765	1270^a^	1203	1883	1778	2319	1865	506	854	3667
KCD 11	1227	2177	1204	2405	1972	1262	1290	1898	1511	2396	2332	750	785	3489
JG 11	1269	2147	993	2282	1528	1032	1122	1606	1489	2049	2272	450	583	3278
SA 1	1500	2027	888	2328	1505	1087	1021	1586	1333	2083	2324	484	611	3389
CD 5%	151	407	148	287	306	215	329	436	385	402	393	257	339	1016
CV %	8	12	9	8	12	13	15	18	12	11	11	20	21	14

*Note*: E1, Bidar; E2, Kalaburagi; E3, Raichur; E4, Siruguppa; E5, Bhimarayanagudi; E6, Hagari.

^a^ Significant performance.

### 3.5 DUS characterization of KCD 11

**FIGURE 5:**
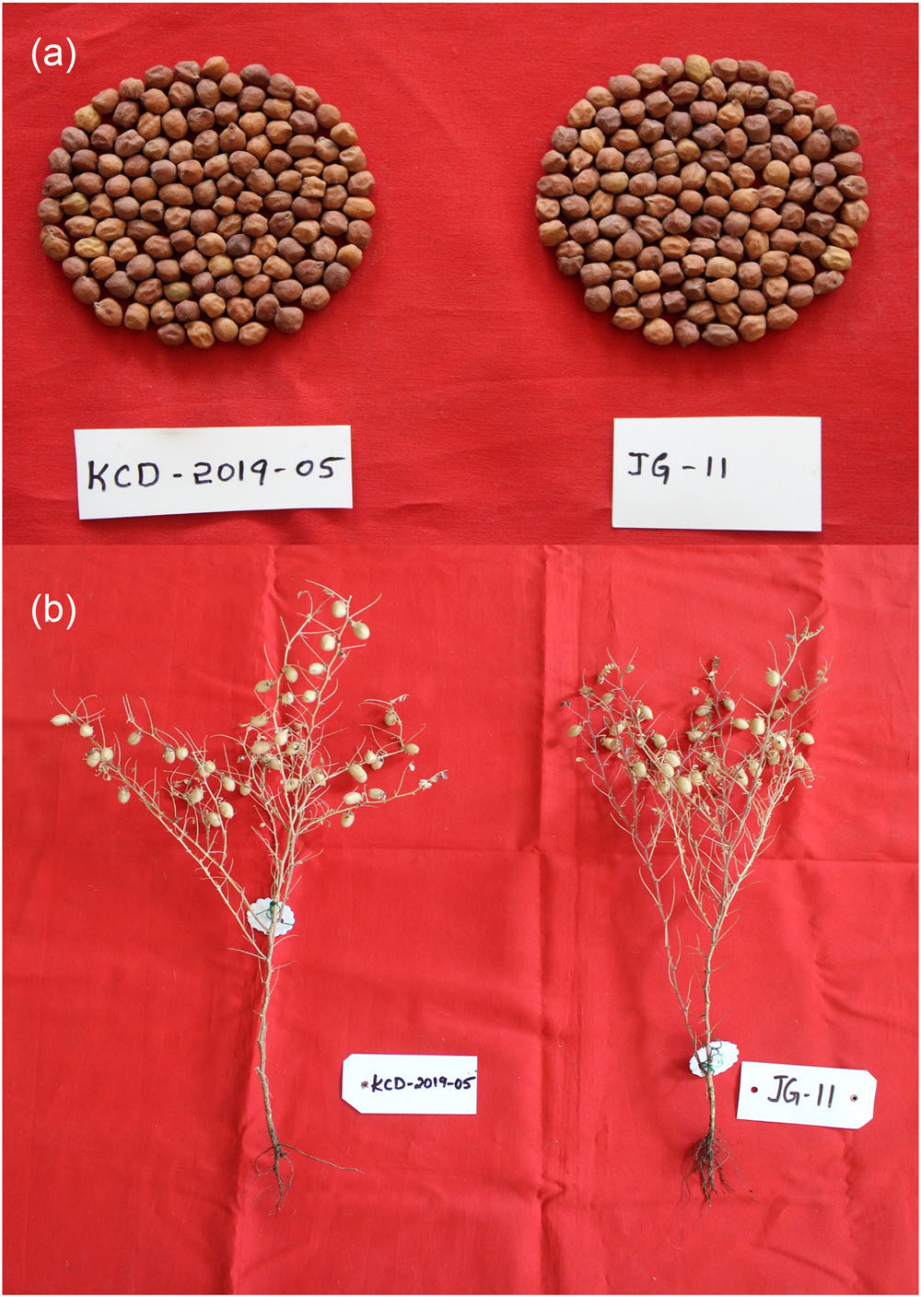
Comparison between KCD 11 (KCD‐2019‐05) and its recipient parent, JG 11. The difference in seed size and the number of pods per plant between KCD 11 and JG 11 is evident. In panel (a), representing the minor differences in seed size and shape between KCD 11 and JG 11. (b), depicting the difference in branching pattern and the count of pods per plant for both KCD 11 and JG 11.

We evaluated KCD 11 for its distinctiveness, uniformity, and stability as per the guidelines of the Protection of Plant Varieties (PPV) and Farmers' Rights Authority (FRA) for chickpea. In this context, we compared KCD 11 with JG 11 and WR 315 for 22 qualitative and quantitative traits (Table 4). When comparing the 100‐seed weight, it was observed that KCD 11 exhibited a higher seed weight than the donor parent WR 315 but was slightly lower (by 1 g) than that of JG 11. The seed size and seed color of KCD 11 were found to be like JG 11 (Figure 5a). Further, KCD 11 showed a greater similarity to the high‐yielding variety JG 11 in terms of yield‐related traits. Nevertheless, the branching pattern and the number of pods per plant were different in KCD 11 compared to JG 11 (Figure 5b). However, no notable variation was observed among KCD 11 and its parental lines for various qualitative traits, including stem anthocyanin pigmentation, plant growth habit, foliage color, leaflet size, leaf pattern, flower color, stripes on the standard, flower/peduncle, peduncle length, pod size, seeds/pod, seed shape, seed color, seed ribbing, seed type, and seed testa (Table 4).

**TABLE 4:** DUS characterization of KCD 11 and its parents using morphological descriptors and quality parameters.

		Parents	
Trait	KCD 11	JG 11	WR 315
Days to 50% flowering	41	41	48
Days to maturity	95	94	97
Plant height (cm)	39	41	37
100‐seed weight (g)	21.7	22.1	12.6
Stem height at initiation of first flower (cm)	28	29	28
Stem anthocyanin	Present	Present	Present
Plant growth habit	Erect	Erect	Erect
Plant: color of foliage	Green	Green	Green
Leaflet size	small	small	small
Leaf pattern	Compound	Compound	Compound
Flower color	Pink	Pink	Pink
Flower stripes on standard	Present	Present	Present
Flower/peduncle	Single	Single	Single
Peduncle length	Small	Medium	Small
Pod size	Small	Medium	Small
Seeds/pod	1	1	1
Seed shape	Angular	Angular	Angular
Seed color	Brown	Brown	Yellow
Seed size	Small	Small	Very Small
Seed ribbing	Present	Present	Present
Seed type	Desi	Desi	Desi
Seed testa	Rough	Rough	Rough

### 3.6 Performance of KCD 11 in AICRP trials

KCD 11 line was forwarded to the AICRP on chickpea for IVT to assess its overall performance and characteristics. In the IVT, KCD 11 was evaluated in two different zones: the south zone (SZ) and the NEPZ, under both irrigated (2021 and 2022) and rainfed conditions (2022). In timely irrigated condition, KCD 11 yielded 1721 kg/ha in the SZ and 1639 kg/ha in the NEPZ. Notably, KCD 11 demonstrated superior performance compared to the check varieties in both zones. For instance, it outperformed JG 11 and Annigeri 1 in the SZ, and in the NEPZ, it surpassed BG 3049, GCP 105, and KPG 59. Specifically, in the SZ, KCD 11 exhibited a 16.5% yield increase compared to Annigeri 1 and a 7.32% increase over JG 11. Within the NEPZ region, KCD 11 exhibited a yield advantage of 9.80% when compared to BG 3049. Moreover, it achieved substantial yield enhancements, surpassing GCP 105 by 13.97% and outperforming KPG 59 by 15.68% (Table 5). Based on its favorable yield performance, KCD 11 was promoted to the next stage, namely, the AVT, specifically in the NEPZ region.

**TABLE 5:** Yield (kg/ha) performance comparison of KCD 11 and other high‐yielding varieties in All India Coordinated Research Project (AICRP) trials conducted across different zones of India.

	NEPZ		SZ		
Variety	2020–2021 (IVT‐irrigated)	2021–2022 (AVT‐1 irrigated)	2020–2021 (IVT‐irrigated)	2021–2022 (IVT‐rainfed)	2021–2022 (irrigated)
KCD 5	1480	−	1581	−	−
KCD 11	1639	1278	1721	1885	2027
JG 11 (C)	−	−	1595	2391	2046
Super Annigeri‐1(C)	−	−	1739	2284	1881
GCP 105 (C)	1410	1647	−	−	−
GNG 2207 (C)	1485	1442	−	−	−
KPG 59 (C)	1382	1678	−	−	−
BG 3043 (C)	1478	1469		−	−

Abbreviations: AVT, advanced varietal trial; IVT, initial varietal trial; NEPZ, north‐east plain zone; SZ, south zone.

## 4 CONCLUSION

Chickpea is an important legume crop in many parts of the world, and it is essential to develop high‐yielding varieties with disease and pest resistance to increase productivity and reduce yield losses. The advanced inbred chickpea line, KCD 11, has shown great promise in multiple trials, consistently demonstrating moderate resistance to FW while maintaining high yield performance across different locations and years. Furthermore, KCD 11 has exhibited superior performance compared to other popular chickpea varieties in Karnataka, establishing its overall superiority. This makes KCD 11 an excellent choice and a valuable addition to the existing range of chickpea varieties, particularly in the SZ, where FW is a major concern. The current study has led to the development of a unique and high‐yielding chickpea variety (KCD 11). Notably, KCD 11 is also recommended for further farm trials to assess its performance. If KCD 11 proves successful in these trials, it has the potential to become a favored option for farmers in the target region. This would contribute to the overall increase in chickpea production and productivity while minimizing yield losses caused by FW.

## AUTHOR CONTRIBUTIONS

**C. Laxuman**: Conceptualization; data curation; formal analysis; investigation; methodology; writing—original draft; writing—review and editing. **Yogesh Dashrath Naik**: Formal analysis; writing—original draft; writing—review and editing. **B. K. Desai**: Resources; writing—review and editing. **Mallikarjun Kenganal**: Investigation; writing—review and editing. **Bharat Patil**: Investigation; writing—review and editing. **B. S. Reddy**: Methodology; writing—review and editing. **D. H. Patil**: Methodology; writing—review and editing. **Sidramappa Chakurte**: Investigation; writing—review and editing. **P. H. Kuchanur**: Methodology; writing—review and editing. **Shiva Kumar K**: Methodology; resources; writing—review and editing. **Ashok Kumar Gaddi**: Methodology; resources; writing—review and editing. **L. N. Yogesh**: Investigation; methodology; writing—review and editing. **Jayaprakash Nidagundi**: Resources; writing—review and editing. **B. M. Dodamani**: Methodology; resources; writing—review and editing. **Gururaj Sunkad**: Methodology; resources; writing—review and editing. **Mahendar Thudi**: Conceptualization; formal analysis; visualization; writing—original draft; writing—review and editing. **Rajeev K. Varshney**: Conceptualization; formal analysis; visualization; writing—original draft; writing—review and editing.

## CONFLICT OF INTEREST STATEMENT

The authors declare no conflicts of interest.

## Supporting information

Supplementary Figure 1: Detection of resistant allele for the SNP marker linked to Fusarium resistance.

## Data Availability

All relevant data are presented in the manuscript.
